# Untapped Potential of Deep Eutectic Solvents for the Synthesis of Bioinspired Inorganic–Organic Materials

**DOI:** 10.1021/acs.chemmater.3c00847

**Published:** 2023-08-18

**Authors:** Marcin Wysokowski, Rachel K. Luu, Sofia Arevalo, Eesha Khare, Witold Stachowiak, Michał Niemczak, Teofil Jesionowski, Markus J. Buehler

**Affiliations:** † Institute of Chemical Technology, Faculty of Chemical Technology, Poznan University of Technology, Berdychowo 4, 60965 Poznan, Poland; ‡ Laboratory for Atomistic and Molecular Mechanics (LAMM), Massachusetts Institute of Technology, 77 Massachusetts Ave., Cambridge, Massachusetts 02139, United States; § Department of Materials Science and Engineering, Massachusetts Institute of Technology, 77 Massachusetts Ave., Cambridge, Massachusetts 02139, United States; ∥ Center for Computational Science and Engineering, Schwarzman College of Computing, Massachusetts Institute of Technology, 77 Massachusetts Ave., Cambridge, Massachusetts 02139, United States

## Abstract

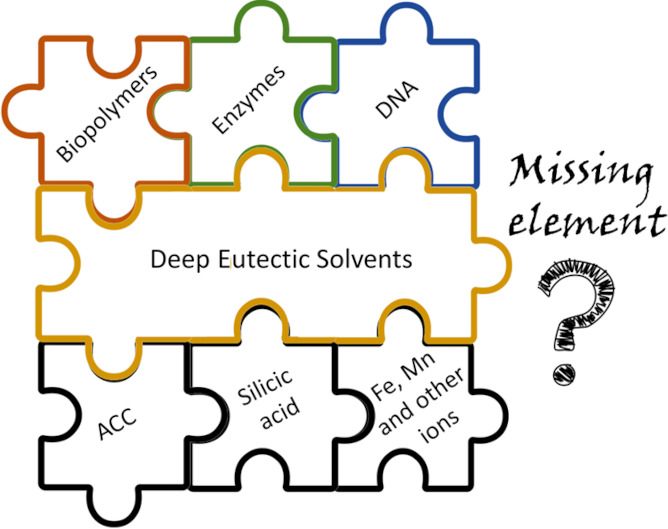

Since the discovery of deep eutectic solvents (DESs) in 2003, significant progress has been made in the field, specifically advancing aspects of their preparation and physicochemical characterization. Their low-cost and unique tailored properties are reasons for their growing importance as a sustainable medium for the resource-efficient processing and synthesis of advanced materials. In this paper, the significance of these designer solvents and their beneficial features, in particular with respect to biomimetic materials chemistry, is discussed. Finally, this article explores the unrealized potential and advantageous aspects of DESs, focusing on the development of biomineralization-inspired hybrid materials. It is anticipated that this article can stimulate new concepts and advances providing a reference for breaking down the multidisciplinary borders in the field of bioinspired materials chemistry, especially at the nexus of computation and experiment, and to develop a rigorous materials-by-design paradigm.

## 1 Introduction

Biomineralization is a highly dynamic fundamental biological process,^1^ by which living organisms create mineral-based (amorphous or crystalline) tissues^2−4^ and is widespread among prokaryotic and eukaryotic organisms. This process is now considered one of the major advances that have critically altered the functional biology, the evolutionary trajectory, and biogeochemical impact of numerous organisms.^5,6^ During 3.7 billion years of evolution, nature has adapted, and often optimized, biomineralized fine structures, which can serve a wide range of functionalities.^7,8^ These functionalities include magnetic navigation,^9^ buoyancy regulation,^10^ mechanical protection/support,^11−14^ light scattering^15^ and transmission,^16,17^ as well as feeding functions.^18−20^ Intriguingly, the biologically formed mineral can be designed for specific functions or in numerous cases have multiple functionalities. Living organisms construct biominerals by utilizing a limited range of substances including polysaccharides (i.e., cellulose and chitin), proteins (i.e., keratin, collagen, or silk), and a few minerals (silica, calcium carbonates, hydroxyapatite, iron oxides). These limited substances are arranged into enormous arrays of complex hierarchical structures.^21^ These examples clearly show that in contrast to many current technologies, natural organisms cannot solely depend on the selection of materials to design a functional system; they instead must generate it from the available existing set of foundational materials, often using a set of principles referred to as the universality-diversity-paradigm (building diverse functions from simple, recurring, and resource-limited building blocks^22^). Eder^21^ further emphasizes that the wide range of properties and functions exhibited by biological materials can be attributed less to their varied compositions and more to the diverse structures they can form. In fact, the multifunctionality and high performance of biomineralized tissues arise naturally from their hierarchical structure (structural organizations that span multiple levels from atomic to the macroscale^21,23−26^), surface morphology, as well as physical and even chemical properties. However, reproduction of the nano- and microstructural features and high degree of hybridization of biomaterials in synthetic materials is not a trivial task. Furthermore, after 50 years of research, current fabrication technologies are still unable to mimic biomaterial fabrication procedures. The mechanistic details underlying biomineralization can serve as inspiration for the development of new material synthesis strategies, such as the use of delicate grain misorientations seen in nature,^14^ and can be exploited as a design principle in synthetic 2D materials.^27^ By applying these principles to more applicable foundational materials, it is possible to obtain novel materials and frequently unforeseen combinations of material properties.^21,24,28^ Therefore, the knowledge that can be extracted from biomineralization is definitively one of the main driving forces for recent progress in biomimetics. As a result, it is on track to becoming a powerful approach in modern materials science, nanotechnologies, and biomimicry.^29−32^

One of the most effective ways to gain an understanding of the basic principles of biomineralization on a molecular level and their application in materials design is a coherent, synergetic approach using explicit reasoning and well-tested principles of multidisciplinary experience, knowledge, and new technologies.^4,33^ Broadly speaking, the influence of chemistry, as a scientific discipline, in the realm of biomineralization can be classified into three main distinct domains:^34^ (i) detailed characterization of the native biominerals encompassing their biochemistry, crystallography, and structural peculiarities; (ii) the design of adequate *in vitro* model systems to answer fundamental questions and verify the hypotheses regarding the possible roles and interactions of organic matrices in controlling crystallization (nucleation as well as crystal growth stages) of inorganics and biomaterial formation; and (iii) the creation of novel synthetic approaches, inspired by bioinspired systems, to regulate crystal morphology, polymorphism, and material characteristics, resulting in the formation of innovative organic–inorganic hybrids.

We believe that by considering the present state of art regarding computational chemistry, machine learning, and artificial intelligence in chemistry, the fourth area should be defined as (iv) utilization of machine learning and artificial intelligence to corelate structure, chemistry, properties, and function to forecast material properties, construct materials from scratch, and uncover new mechanisms beyond intuition.^35^

Paraphrasing Cölfen, “A crystal-clear view”,^36^ we fully agree that “with the biomineralization strategies in the hands of synthetic chemists, the formation of hierarchically structured organic–inorganic composite materials with enhanced material properties will conceivably have the potential to outperform biominerals because of the larger range of materials/solvents and advanced equipment available to synthetic chemists”.^36^ In fact, the concept of creating bioinspired hybrid materials with advanced properties from fundamental building blocks remains a largely uncharted area in materials science. Currently, much attention is paid to developing bottom-up approaches and soft chemistry methods targeting the synthesis of hybrid materials inspired by biomineralization processes.^37^ Mimicking of natural biominerals or biomineralization as a phenomenon facets several challenges that include solubilization of biopolymers (chitin, common component of biominerals, is almost insoluble in most solvents) or stabilization of inorganic precursors (i.e., silicic acid) that are crucial to achieve mineral deposition on all levels of structural hierarchy. All these challenges can be effectively solved by deep eutectic solvents (DESs).

DESs are known to be “green”, safe, and inexpensive mixtures and can provide tremendous opportunities as reaction media for modern bioinspired chemistry. These designer mixtures can serve as structure directing agents, protein and inorganic stabilizers, and polymer solvents. All these mechanisms can be applied simultaneously during the development of biomineralization-inspired materials. Therefore, the aim of this article is to highlight the unexplored potential of DESs as a reaction medium for the synthesis of biomineralization-inspired inorganic–organic hybrid materials, including computational design. Surprisingly, despite the wide application of DESs as (i) a medium in the synthesis of inorganic materials and (ii) solvents for biopolymers, this unconventional approach has not been studied in fine detail from the point of view of bioinspired materials chemistry. Due to the preorganized, supramolecular nature of deep eutectic solvents (DESs), they offer a remarkable soft template for directing the creation of biomineralization-inspired hybrids on a spectrum ranging from the atomic to the macroscopic scale. Additionally, the compositional flexibility of DESs lends to limitless compositions and, in turn, an enormous range of attainable properties. Utilization of the properly selected DES combined with the truly multidisciplinary and multiscale understanding of the impact of their structural peculiarities on the properties of biomacromolecules (proteins, enzymes, or polysaccharides) and inorganic building blocks will be game-changing in terms of biomineralization-inspired hybrids. This sustainable approach opens tremendous perspectives and could lead to the next generation of bioinspired hybrids with sophisticated features that can outperform the natural biominerals. We expect that there is a significant potential for fundamental research in exploring the application of DESs in contemporary biomimetics. This article will encourage the scientific society to undertake research that will seek to answer the question: How far can deep eutectic solvents push the boundaries in bio-inspired materials science?

## 2 Deep Eutectic Solvents: From Chemistry to Bioinspired Synthesis

**Figure 1:**
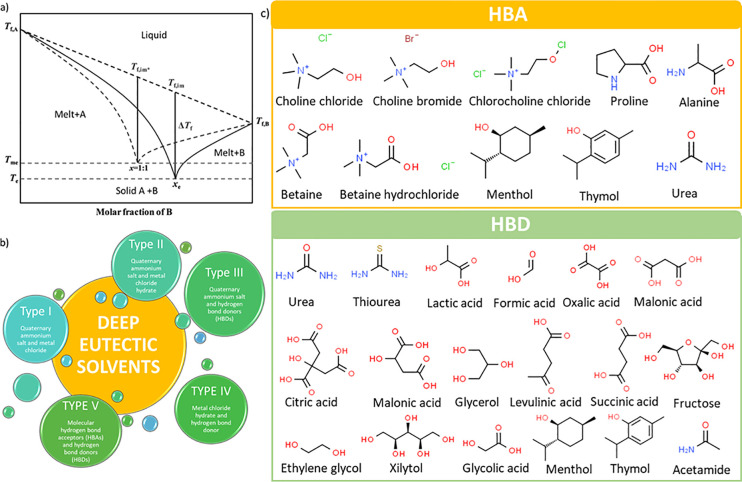
(a) Example of DES phase diagram (*T*_f,A_ and *T*_f,B_, melting points of individual components A and B; *T*_f,im*_ and *T*_f,im_, ideally interpolated temperature at the assumed maximum and the practical eutectic composition; *T*_me_ and *T*_e_, estimated maximum and the practical eutectic temperature; and *x*_e_, the eutectic composition). Reproduced from ref (65). Copyright 2020 American Chemical Society. (b) Diversity of DESs according to Smith et al.^39^ and Hansen et al.^40^ (c) Commonly exploited HBAs and HBD in DESs research.

The term “deep eutectic solvent” first appeared in academic literature in 2003, when Abbott et al.^38^ discovered mixtures of choline chloride and urea with significantly reduced melting points compared to their individual pure components.^39^ Significant research into the chemistry of DESs has resulted in several changes over time in their definition. To present knowledge and within the context of this article, DESs are defined as mixtures composed of two or more components, which can self-associate to create a new eutectic phase characterized by a significantly reduced melting point (usually below the room temperature) in comparison with the melting points of their individual pure components^40−46^ (Figure 1a). DESs maintain the identities of their components, which interact via hydrogen bonding, as opposed to covalent bonding. Further, DESs remain in a liquid state: molecular dynamics simulations^47^ and inelastic neutron scattering studies^48,49^ revealed that the strength of hydrogen bonds between DES-forming components is weak enough to prevent them settling into a cocrystal.^50^ So far, depending on the chemical composition, five different categories of DESs are distinguished (Figure 1b). Accordingly, to Hansen et al., these categories are briefly defined as follows: type I, constituted by a quaternary ammonium salt and a metal chloride; type II, constituted by quaternary ammonium salt and a metal chloride hydrate; type III, constituted by a quaternary ammonium salt and a neutral species that acts as a hydrogen bond donor (HBD), typically an organic molecular entity like an amide, carboxylic acid, or polyol; type IV, formed by a metal chloride hydrate and HBD; and type V, represented a recent category exclusively comprised of nonionic, molecular hydrogen bond acceptors (HBAs) and hydrogen bond donors (HBDs), that opens new avenues for the design of hydrophobic DESs.^51−53^

Type III DESs have been the focus of extensive research because of (i) low melting points, which exhibit lower melting temperatures compared to other types of DESs; (ii) composition flexibility, which can be largely designed and customized to achieve DESs with a wide range of physicochemical properties; and (iii) application potential, which has shown promise in a wide range of applications and as a replacement to traditional solvents in numerous industries.

It is worth emphasizing that although type III DESs have received considerable focus, investigations into other DES types, including type I and type II, greatly contribute to the overall advancement and comprehension of deep eutectic solvents. However, recent reports on the eutectic mixtures that fit the definition of DES, but do not fit easily into these five categories, suggest that other types have yet to be discovered.^40^

DESs have also been described differently throughout literature. Mixtures obtained from natural components are classified as NADES (natural deep eutectic solvents).^54−56^ Recent reports about new DES subtypes include active pharmaceutical ingredient-DES(API-DES)^57^/therapeutic-DES (THEDES),^58−61^ which refer to a combination of two constituents that, at a specific molar ratio, transform into a liquid state at room temperature and in which one of them is an active pharmaceutical ingredient. Additionally, magnetic deep eutectic solvents (MDES) are considered a special category of conventional deep eutectic solvents.^62−64^ MDES are defined as mixtures comprising two or three components, which include a HBA, HBD, and an additional magnetic component such as a metal chloride.^64^

We believe that recent identification of DESs in extremophilic organisms^66^ is an important historical landmark that will lead to discoveries of new DESs classes. Research on DESs has exploded rapidly in recent times, both in the context of fundamental and applied research.

So far, large numbers of excellent critical review articles^39,40,43,67,68^ and books^69−71^ describing the chemistry of DESs as well their practical applications in energy storage,^72^ catalysis,^73−75^ analytical chemistry,^76−78^ extraction^79−82^ or separation,^81^ environmental monitoring,^83^ CO_2_ capture,^84−86^ biotechnology,^87^ biocatalysis,^88,89^ drug discovery and delivery,^90,91^ materials science,^92−94^ and biopolymer processing^67,95−97^ appeared in the past few years. In sum, this work is focused only on the properties that are crucial from the biomineralization-inspired point of view, including biopolymers processing, self-assembly, and inorganic synthesis.

**Figure 2:**
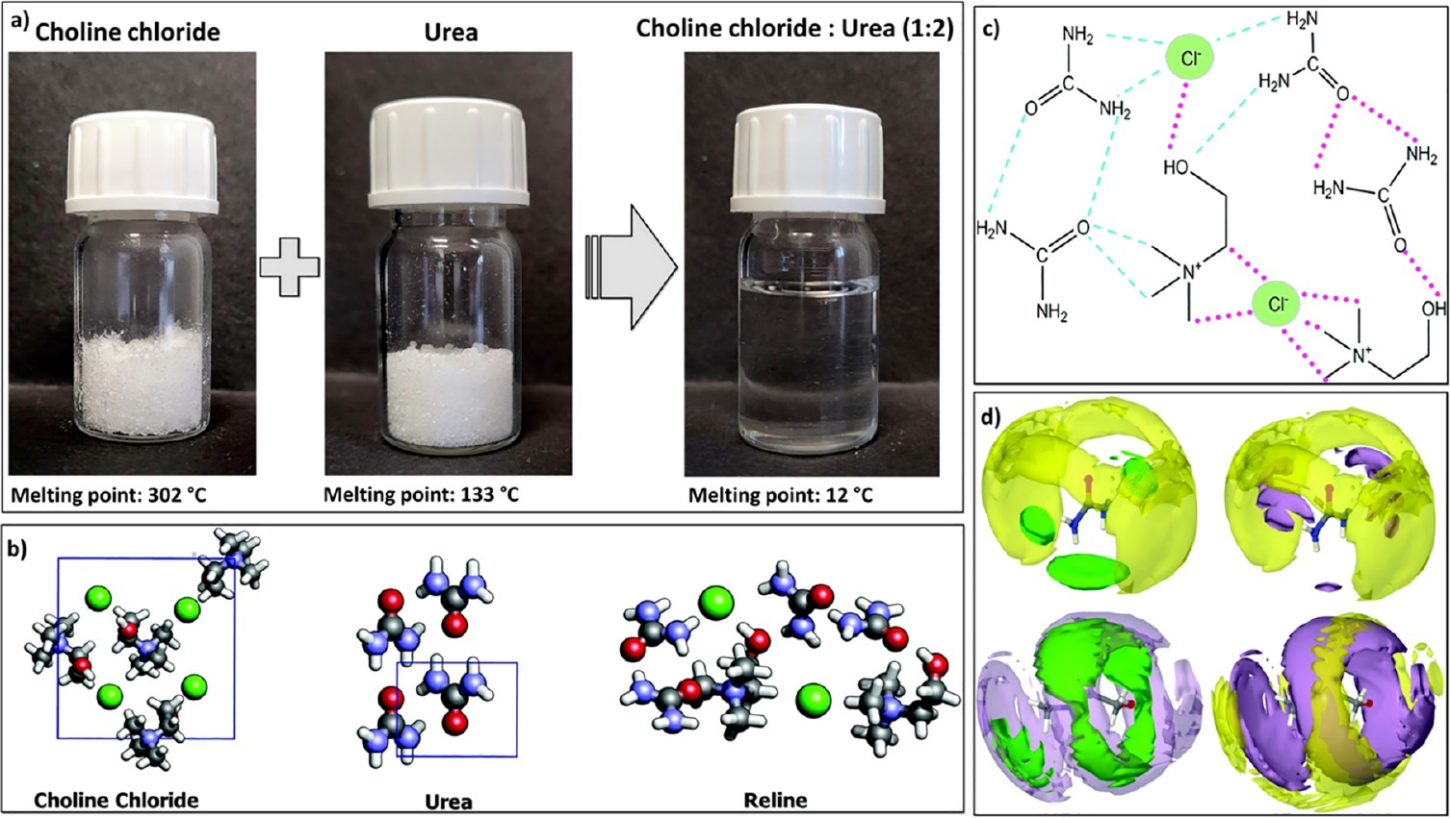
(a) Schematic representation of the preparation of choline chloride/urea-based DES and differences in the melting points. (b) Molecular representation of the crystal lattices of choline chloride and urea, together with the model of reline (choline chloride:urea, 1:2) after geometry optimization. Adapted with permission from ref (127). Copyright 2017 Royal Society of Chemistry. (c) Schematic representation of the choline chloride:urea cluster with the intermolecular interactions in the formed DES (dashed lines) and those already present in the pure solids (dotted lines). Adapted with permission from ref (127). Copyright 2017 Royal Society of Chemistry. (d) Spatial density functions demonstrate the probability of the 3D structures of the various parts of (choline chloride:urea, 1:2). Reprinted with permission under a Creative Commons Attribution (CC-BY) 3.0 from ref (128). Copyright 2016 Royal Society of Chemistry.

Typical DES synthesis methods are relatively simple and fast as they are based on heating and stirring the constituents together until a homogeneous liquid is formed (Figure 2). This makes DESs extremely interesting and desirable alternatives to conventional organic solvents and ionic liquids (ILs). There are countless possible combinations and ratios of HBAs and HBDs that can constitute DESs, and these combinations can be finetuned to exhibit desired properties. Further, it is predicted that DESs can be successfully adapted for various applications, including dissolution and processing biopolymers,^98^ inorganic synthesis,^99^ or 3D printing.^100^ Alongside the aforementioned reasons, low costs and low maintenance make DESs excellent candidates for utilization on the industrial scale, which is the main barrier in the case of most ILs.^101−104^

However, such a tremendous level of tunability of DESs is also considered an impediment that requires significant resources, including time to allow for the discovery of mixtures with beneficial or desired performance. In effect, the scientific community is focusing on QSAR (quantitative structure–activity relationship) and QSPR (quantitative structure–property relationship) models that in the future will be able to predict and select the most promising DES, thus eliminating the need for hundreds of hours of expensive laboratory work.^105^

Various physicochemical properties of DESs have been studied and described lately because such data is considered crucial in terms of developing known and seeking new fields of their application. Taking into consideration that DESs’ properties can alter significantly at different compositions, analyzing not only the eutectic composition but also other combinations is often considered a reasonable solution. However, an analysis of the literature indicates that studies are most often conducted for one HBA to HBD ratio, which is likewise not a eutectic point (which refers to composition characterized by the lowest melting point). This fact significantly hinders the discovery and indication of favorable physicochemical parameters from the application point of view. Within the framework of discovery potential, modeling plays a crucial role, including quantum mechanical^106,107^ and deep learning approaches.^108−117^ Specifically, a new generation of generative language models can provide powerful avenues to explore the potentiality of DES structures that have not yet been discovered, specifically focused on *de novo* design.

The following subsections of this article will provide a general overview of the properties of DESs and the role these properties play in the context of biomineralization-inspired materials.

### 2.1 Lowered Melting Point

One of the key parameters determining the usefulness of DESs in various applications is its ability to lower the melting point. As was established by Abbott et al.,^118^ the combination of choline chloride (*T*_m_ = 302 °C) with urea (*T*_m_ = 133 °C) in a ratio of 1:2 leads to a DES that is a liquid at room temperature (*T*_m_ = 12 °C). This crucial discovery showed that DESs can be successfully applied as excellent solvents for processes conducted even at room temperature. Soon after, it was noticed that the melting point of the formed DES varies greatly and depends on the chemical structures of both the HBA and HBD, (e.g., choline chloride:thiourea (1:2) has *T*_m_ = 69 °C, choline chloride:1,3-dimethyl urea (1:2) has *T*_m_ = 70 °C, choline chloride:ethylene glycol (1:2) has *T*_m_ = 66 °C, choline chloride:glycerol (1:2) has *T*_m_ = −40 °C, choline fluoride:urea (1:2) has *T*_m_ = 1 °C, ZnCl_2_:ethylene glycol (1:4) has *T*_m_ = −30 °C, and H_2_O:DMSO (1:4) has *T*_m_ = −69 °C).^40,68,119,120^

Conducting processing at ambient temperatures can contribute to strengthening the hydrogen-bonding accepting ability, which theoretically improves the ability to dissolve biomass, such as cellulose, starch, or chitin. Typically, dissolution processes are performed at temperatures ranging from 50 to 100 °C, due to high viscosity of DESs.^121,122^ This means that both the composition and ratio between HBA and HBD in DESs must be properly selected so that the obtained system is in a liquid state under the assumed conditions. As was reported previously, exposition of DESs consisting of choline chloride and urea on the temperature above 120 °C for a longer period of time leads to decomposition of urea, which can be successfully applied for synthesis of novel inorganic materials, like MnCO_3_,^123^ [NH_2_(CH_3_)_2_]_2_Sn_3_Se_7_·0.5NH(CH_3_)_2_, [NH_4_]_2_Sn_4_Se_9_ and [NH_4_]_3_AgSn_3_Se_8_,^124^ (VO)_2_P_2_O_7_ and VOPO_4_,^125^ or Zn(O_3_PCH_2_CO_2_)]·NH_4_.^126^

On the contrary, utilization of DESs at lower temperatures can lead to formation of various inorganic compounds exhibiting unique characteristics, such as nanofibrous networks of RuCo_2_O_4_,^102^ (N-doped) ceria nanoparticles, calcium phosphate nanoparticles,^129^ or NiCo_2_O_4_ nanooctahedrons.^130^

Interestingly, low melting points of DESs obtained from choline chloride and ethylene glycol^131,132^ or polymerizable HBD, like acrylic acid,^133^ maleic acid, and acrylamide,^134^ were successfully utilized for fabrication of 3D-printed conductive organogels, which have proven to be attractive sensitive strain sensors.

### 2.2 Intramolecular and Intermolecular Hydrogen Bonding

It is assumed that the sum of interactions and effects, originating from the unique structures of both HBA and HBD are responsible for achieving the depression of melting temperature in DESs. Besides the steric effects related to the spatial arrangement of DES components, hydrogen bonds between the HBA and HBD are believed to play a major role, which contributes to weakening ionic attractions between cation and anion in HBA.^118,135−137^ Moreover, other forces, particularly van der Waals interactions, were recognized as particularly important in terms of physicochemical properties of DESs, since they can significantly influence the mobility of molecules incorporated into the eutectic system.^138,139^ This underscores the general relevance of H-bonding for biological and bioinspired materials.^140−144^

Interestingly, the strength of hydrogen bonding between HBA and HBD in various binary DESs has been correlated to its capability to extract chitin from crustacean waste. A purity level of 93% was attained for chitin extracted by DES consisting of choline chloride and formic acid (1:2).^145^ These findings are also supported by the fact that DESs of choline chloride and ethylene glycol, or glycerol, were found to be ineffective for dissolving this biopolymer, whereas DES compositions of choline chloride and urea or thiourea achieved satisfactory solubility at a level of 8–9%. Interestingly, these DESs were more effective than compositions comprising various dialkylimidazolium halides or betaine hydrochloride, which suggests that the structure of HBA could significantly influence the process of chitin dissolution.^146^ Moreover, the presence of imidazolium rings in this case hindered the efficacy, which contrasts with previous reports demonstrating their excellent polysaccharide-dissolving ability.^147^

It has been found that very strong hydrogen bond interactions in DESs facilitate their homogeneous dispersion and help to obtain a stable morphology of various inorganic nanomaterials.^102^ For instance, Zhang et al.^148^ stated that since the chloride ion was incorporated into hydrogen bond networks in DESs, it has a structure-directing ability, which induced the morphology of fabricated advanced nano electrocatalysts consisting of NiCo_2_O_4_. The crucial role of hydrogen bonding in acceleration of the polymerization rate was noticed during the fabrication of 3D-printed ultratough transparent conductive elastomers via stereolithography. The preparation strategy enabled the acceleration of the photopolymerization rate of a mixture consisting of maleic acid:choline chloride and acrylamide:choline chloride and the rise of the double bond conversion. Since choline chloride can form hydrogen bonds with both HBDs, the mixed solution before polymerization was transparent, contributed to the formation of uniform polymers.^149^ As demonstrated by Cai et al., the utilization of acrylic acid as HBD leads also to transparent and uniform materials, which was confirmed by analysis of the collected optical images.^133^

### 2.3 Thermal Stability/Volatility

DESs are often compared to ILs because they share many of the same properties, e.g., generally high thermal stability and low volatility because of strong interactions between HBA-HBA, HBD-HBD, and HBA-HBD. These properties are considered especially desirable for possible replacement of volatile organic compounds (VOCs) currently used as solvents in industry.^150^ It should be noted that the thermal stability of DESs limits the maximum operating temperature, in which they can be effectively applied. In accordance with the literature, while the vapor pressures of DESs are multiple times lower than that of common VOCs (e.g., hexane or acetonitrile), they were simultaneously found to be significantly greater compared to ILs equivalents.^40^ These dependencies were described in more detail by Ravula et al.,^151^ who ranked the following fluids with increasing volatility: dicationic ILs < aprotic ILs < polymeric ILs < protic ILs < DESs < long-chain PEGs < triglyme, short-chain PEGs < VOCs.

On one hand, the high thermal stability of DESs allows their use at higher temperatures, which can significantly increase the reaction rate. Furthermore, the solvent-dissolving ability generally notably increases at higher temperatures, due to the reduction of viscosity and surface tension, which eventually contribute to enhanced diffusivity.^152,153^ On the other hand, high volatility or low stability of some DESs constituents can alter their composition or HBA:HBD ratio, which significantly reduces their application potential. Particularly, the nonionic nature of most common HBDs makes them more susceptible to decomposition or evaporation compared to ionic HBAs. For instance, at around 125 °C, urea degrades rapidly, while ChCl begins to decompose at about 225 °C.^154^ Other examples confirm that tendency, e.g., thiourea, menthol, and acetamide decompose at 110 °C,^155^ 181 °C,^156^ and 188 °C,^157^ respectively.

As DESs represent mixtures rather than pure chemical compounds, thermogravimetric analysis (TGA) could only provide data regarding “apparent” vapor pressures, mainly because of plausible preferential species volatilization leading to change in the composition of the analyzed mixture. This phenomenon is particularly valid for DESs obtained from compounds that are more susceptible to evaporation or sublimation than decomposition, like menthol, thymol, or coumarin.^55^ Analysis of literature data leads to the conclusion that thermal stability of DESs depends on the stability of both ingredients used for their preparation as well as on the mutual interactions between them (mainly hydrogen bonds network).

### 2.4 Viscosity

Viscosity, as one of the most essential parameters describing DESs, represents the resistance to deformation under the influence of shear forces. Extensive hydrogen bond networks occurring between constituents of DESs together with others (ionic and van der Waals interactions) significantly affect the mobility of ions/molecules and, as a result, DESs are usually characterized by considerably high viscosities, usually exceeding 50 mPa s. Generally, at ambient temperature this parameter varies from 2.6 mPa s for nonionic DES, menthol:dichloroacetic acid (1:2), to more than 80 000 mPa s for ionic–ionic DES, choline chloride:ZnCl_2_ (1:2). Detailed values have been summarized previously and can be found elsewhere.^40,158−161^ It was established that higher viscosities of DESs containing ionic moieties can be attributed to the Coulombic forces between the cation and anion in HBA.^162^

As was previously stated, viscosity is directly associated with transport properties within the liquids phase, such as diffusion of compounds dissolved in a DES. Therefore, analogously as in the case of ILs, much effort is being devoted to reducing the viscosity of DESs to increase their efficacy as working media in the dissolution of biopolymers, synthesis of inorganic, and organic compounds as well as sophisticated hybrid nanomaterials.^163^ To achieve that goal, various strategies have been elaborated, such as the following: (i) Selection of appropriate HBA: due to strong van der Waals interactions, viscosity generally increases as the alkyl chain in HBA increases, therefore short alkyls contribute to improvement in the mobility of DES constituents.^160,164^ (ii) Selection of appropriate HBD: due to the fact that a strong hydrogen^165^ network leads to a rise of viscosity, compounds with fewer functional groups able to form H-bonds are preferable (this fact explains extremely large viscosities of sugar-based DESs that exceed 10 000 mP s even at elevated temperatures). (iii) Addition of water: it is beneficial for significantly reducing hydrogen-bonding interactions, which subsequently leads to a decrease in viscosity. In the case of some hydrophobic DESs, viscosity reduction can be achieved by addition of organic solvents (methanol, acetone, or DMSO). (iv) Increase in temperature: similarly as in the case of ILs, DESs demonstrate an exponential decrease in viscosity with a rise of temperature, which can be described by the Arrhenius model:  is the activation energy for viscous low, *A* is a cofactor, and *R* is the molar gas constant. The nonlinear characteristic of this relationship indicates that even a slight increase in temperature can contribute to substantial reduction of viscosity. It should also be noted that reduction of viscosity with increasing temperature is more pronounced in the case of DESs compared to conventional VOCs.^166,167^ (v) Addition of third component in the mixture: depending on the type of DES, this action can decrease the overall viscosity (e.g., for ionic–ionic DES 1-butyl-3-methyl-imidazolium:ZnCl_2_ addition of acetamide reduces viscosity 3–5 fold; for ionic-nonionic DES choline chloride:xylitol addition of glycerol reduces viscosity 2–3 fold) or can increase it (e.g., for ionic-nonionic DESs choline chloride:urea and choline chloride:malic acid addition of glycerol provides a rise of viscosity by at least 50%).^165,168,169^

Excessive viscosity not only impedes the mixing and separation processes carried out in DESs but also influences the dynamic interaction between heat and substances, thereby severely restricting their extensive practical use. Despite utilization of cost-effective substrates and facile method of preparation of DESs, their high viscosity is still often considered as the greatest disadvantage and impediment to their commercialization; therefore, all studies focused on overcoming this important issue are strongly justified.

### 2.5 pH

Depending on the chemical structure of the starting materials, the obtained DESs can be available in a wide range of pH values. DESs obtained from neutral compounds, like choline chloride and sugars exhibit a neutral pH, whereas compositions containing polyols and organic acids can be characterized by acidic behavior.^165,170,171^ On the other hand, urea and potassium carbonate act dominantly over choline chloride and glycerol which contribute to the basic nature of such DESs.^172^ Literature surveys^171,173^ indicate also that Kamlet–Taft solvatochromic parameters are being determined to describe the character of the obtained DESs. Hence, the calculated parameters α, β, and π* are associated with hydrogen-bond acidity (hydrogen bond donating strength), hydrogen-bond basicity (hydrogen bond accepting strength), and dipolarity/polarizability of the solvents, respectively. Interestingly, the value of the Kamlet–Taft β parameter is positively correlated with the rate of dissolution of polysaccharides in DESs. In the case of chitin, a relatively acidic environment was more favorable for the extraction of chitin from crustacean waste. Additionally, it was discovered that purity of chitin is positively correlated with the HBD acidity. According to another study, pH values had a measurable effect on the extraction efficiency of the proteins with the use of binary and ternary DESs, thus showing the importance of this parameter also in separation processes.^174^ Moreover, the stability of various enzymes, like lipase is also correlated with DESs’ hydrogen bond acidity, which in the future can be utilized in synthesis of novel materials via biocatalysis.^175^

### 2.6 Density

Another critical parameter of DESs in various applications is their density. Density is utilized in the development of various thermodynamical models, process simulations, and engineering estimations that are required for studying fluid flow, mass transfer, heat transfer, and reaction kinetics of DES. State-of-the-art indicates that the most of the described DESs are characterized by higher densities compared with water, with values ranging from 1.0 to 1.6 g cm^–3^ at 25 °C.^176,177^ Nonetheless, some exceptions can exhibit even greater densities (FeCl_3_ 6H_2_O:glycine (2:1) = 1.677 g cm^–3^) or extraordinarily low densities (borneol:thymol (1:2) = 0.800 g cm^–3^).^178^ It was also noted that densities lower than water can be obtained for hydrophobic DESs, which generally contain structurally more bulky constituents, and consequently, the packing fraction of atoms is strongly reduced.^179,180^ Generally, the density of DESs decreases linearly with increasing temperature, in addition to relying on the presence of vacancies in the DES network.^176,181^ Previous studies have extensively documented the densities of known DESs.^40,158,161,177,180,182−191^ In recent years, many computational methods have been developed to predict with high accuracy the density of DESs using the classical quantitative structure–property relationships (QSPR) approach with suitable mathematical equations,^183,185,192−194^ artificial intelligence,^186^ and machine learning.^180,184,195^ Interestingly, Alkhatib et. al^196^ and Kovács et. al^54^ described state-of-the-art, perspectives and guidelines on modeling the physicochemical properties of DES, including density. With such a substantial number of models, model performance can be compared with, e.g., Tak et al.,^183^ who compared results calculated from five of the existing models with experimental results to reveal the one that is the most precise. Researchers noted also that models based on the cohesion factor of the cubic equation of state (predictive Soave–Redlich–Kwong (PSRK) equation of state with NSM1 alpha function model (NM-PRNSM1), PSRK equation of state with original derived alpha function model (NMSRK))^197,198^ are able to compute density with higher precision compared to models based on correlations like the Reid (RR) model, McHaweh (MH) model, and linear generalized model (LGM).^199^ The high level of scientific interest in computer-based predictive methods clearly demonstrates that further progress in this field is inevitable and will help to increase in understanding of the interactions occurring between the respective components of DESs.

### 2.7 Conductivity

**Figure 3:**
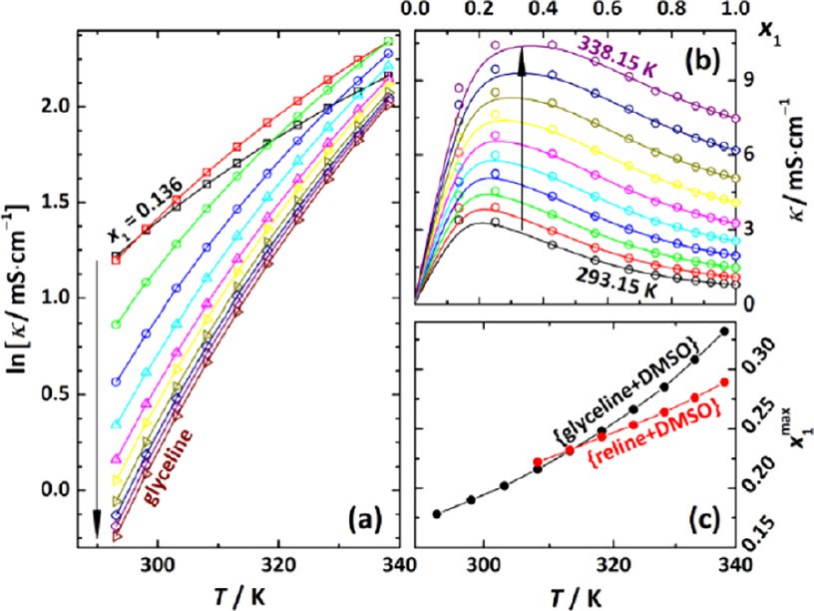
Electrical conductivity as a function of (a) temperature and (b) glycine mole fraction. (c) Temperature dependence of the composition (*x*_1_^max^) of maximum conductivity obtained for glyceline and reline. Reproduced from ref (208). Copyright 2021 American Chemical Society.

Electrical conductivity is a fundamental physical property that represents how easily a material can conduct electrical current. Nowadays, it is also considered to be the most important factor for the design, control, and optimization of electrochemical processes.^200^ For example, in the manufacturing of bioinspired materials made of ultrathin nanofibers by an electrospinning technique, conductivity of solvent is considered to be a crucial parameter affecting the course of the process and the morphology of the resulting fibers.^201,202^ Additionally, electrical conductivity provides essential information for designing the cathodic protection required to prevent corrosion.^200^ When measured in a broad temperature range, the conductivity of DESs usually exhibits non-Arrhenius behavior, typical for glass-forming liquids.^203^ DESs and their solutions are usually characterized by conductivity between 0.01 and 30 mS cm^–1^. These values are higher than pure solvent conductivity (0.0001–0.0050 mS cm^–1^ for distilled water) but similar to ILs (0.01 to 100 mS cm^–1^) or nonaqueous electrolytes (around 10 mS cm^–1^ for Li-ion electrolytes) and lower in comparison with aqueous electrolytes (50 to 250 mS cm^–1^).^39,200,204−206^ In accordance with many reports, the conductivity of DESs rises with an increase of the temperature as well as the amount of water, which is generally a decrease in viscosity of tested compositions.^68,170,181,207^ Interestingly, the electrical conductivity of DES–solvent systems generally show a maximum value at a relatively low DES concentration (Figure 3).^208,209^

These observations imply that DESs systems are unique compared with either organic solvent mixtures or aqueous electrolyte solutions, in which concentration and conductivity are often directly proportional to each other.^209^ Recently, promising attempts have been made to build computer models based on multiple linear regression or using artificial neural networks to predict the conductivity of DES.^170,200,210−212^ However, it should be noted that these models are based on a relatively small amount of data, which limits their applicability. It can be assumed that in the coming years, with more data and use of DESs, these models will be significantly improved.

### 2.8 Polarity

The most widely applied method to describe polarity properties of organic compounds, including DESs and ionic liquids, is the Kamlet–Taft empirical polarity scale. To capture the complexity of interactions, this scale, based upon linear solvation energy relationships, is composed of the complementary scales of hydrogen bond acidity (α), hydrogen bond basicity (β), and dipolarity/polarizability effects (π*). Generally, polarity is associated with multiple overlapping elements, such as the hydrogen-bonding, electron pair donor–acceptor interactions, Columbic interactions, and various dipole interactions. This parameter is rather complex as shown in solvent-dependent phenomena, e.g., the rate and selectivity of many chemical reactions in DESs, interpreting the interaction of DESs with various organic and inorganic materials as well as biomass.^213−215^ Moreover, since the polarity increases with an increase in the intermolecular interactions, this parameter is often described as a unique solubilization property of a solvent, which is crucial in studies focused on utilization of DESs as a “green” alternative to common solvents.^161^ The solvent polarity, separately or together with hydrophobicity and hydrogen bond characteristics, can also affect the extraction capacity or protein stability and activity. These findings clearly indicate the need for conscious selection of a specific HBA:HBD pair, which should certainly be based on the current state of knowledge in a specific application.^189,216^

The role, significance, and dependencies in all three parameters α, β, and π* has been thoroughly outlined in other insightful review articles.^40,68,161,176,214^ On the basis of the above mentioned reports, the following polarity of DESs can be arranged in the following order: monoacid-based hydrophilic DESs < ionic or neutral hydrophobic DESs < diacid–based hydrophilic DESs < alcohol-based hydrophilic DESs < water. The HBD structure and water content have also been shown to affect the polarity of the systems. Simple replacement of the HBD or change in the water content can significantly alter solvent polarity, likewise with just water or methanol.^215,217^ Interestingly, results summarized by J. Cao and E. Su^161^ indicate also that the polarity of hydrophobic DESs mainly depends on the specific properties of HBA that originate from its chemical structure.

### 2.9 Surface Tension

Surface tension is a measure of cohesive forces in liquid on the surface and plays a pivotal role in diverse processes relying on wetting, permeability, lubrication, and bubbling. Therefore, it is considered a key physicochemical property for the application of DESs in the field of interface and colloid processes,^218−220^ including biomineralization-inspired material syntheses.^221,222^ It has been proved that the solution surface tension plays a significant role (besides the already identified parameters, such as the biopolymer functional groups and the biopolymer concentration) in the crystallization of biominerals.^221^

There are few reports discussing the influence of this parameter on application DESs in other important areas, like biopolymer processing or synthesis of various organic/inorganic materials. The majority of DESs are characterized by surface tension (γ) between 30 and 70 mN m^–1^ in standard conditions,^219^ and the stronger hydrogen-bonding interactions usually lead to a higher value of this physicochemical property (e.g., for choline chloride:lactic acid (1:2) γ = 47.4 mN/m, for betaine:lactic acid (1:2) γ = 46.8 mN/m, for choline chloride:glycerol (1:2) γ = 57.8 mN/m).^219^ Interestingly, the temperature and the molar ratios between the components are among the primary factors that strongly affect γ and can be easily adjusted.^218^ Similarly, to traditional VOCs or ILs, the γ of DES linearly decreases with increasing temperature.^176,219^ Obviously, γ in DES can be lowered by addition of surface-active compounds, which possess amphiphilic structures.^223^ Interestingly, the presence of crystal water in HBAs would contribute to a slight decrease in the surface tension. However, the presence of a small amount of DES significantly decreases the surface tension of water.^219^ Additionally, due to strong hydrogen-bonding interactions, small addition of solvents like alcohols, acetone, or ethyl acetate can lead to decrease in surface tension of DES; nevertheless, a small addition of DES to these solvents does not affect their surface tension.^219^ There are many studies focused on investigation of the surface tension of DESs based on which of several predictive models were created.^218^ It should be stressed that the most general surface tension and density models developed by Haghbakhsh et al.^218^ allow us to predict these properties at various temperatures, even for novel, not yet discovered DESs. This area needs to be further explored and related to the biopolymer processing and formation of biopolymer-mineral hybrid materials.

### 2.10 Self-Assembly

**Figure 4:**
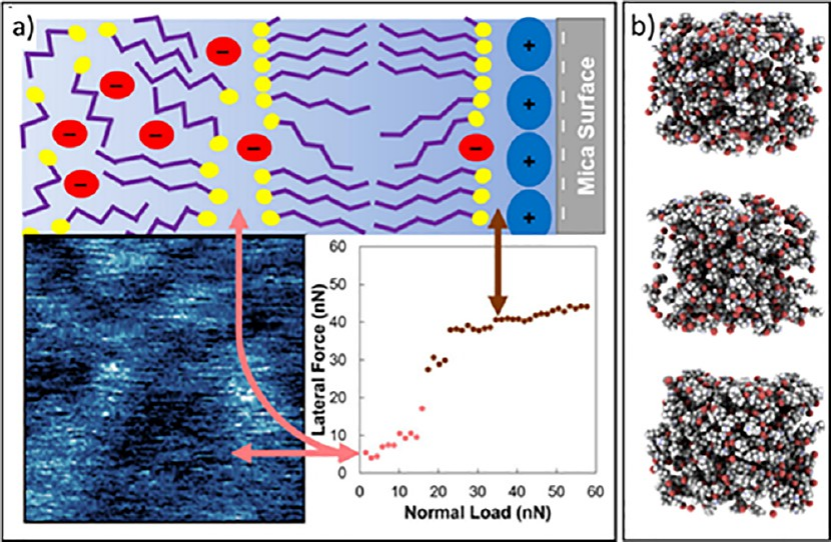
(a) Representative schematic of the transition from the multilayer regime to the boundary regime for ChCl/C_6_OOH on mica. Reprinted with permission from ref (226). Copyright 2022 Elsevier. (b) EPSR model snapshots of nanostructured propylammonium bromide:glycerol (1:2). Adapted from ref (225). Copyright 2018 American Chemical Society.

It is hypothesized that the DESs generally do not contain amphiphilic molecules or ions and therefore lack the beneficial self-assembled nanostructures found in numerous ILs. On the other hand, formation of intra- and intermolecular interactions, including Coulomb forces, hydrogen bonding, van der Waals interactions, electrostatics, dispersion forces, and a polar–polar segregation in DESs dictates their distinct bulk liquid and interfacial nanostructure.^224^ DES nanostructures demonstrate different spatial arrangements of the distinct species and exist over several length scales, from molecular- to nano- and mesoscales.^224^ The nanostructure of DESs is inspiringly discussed in a review by Bryant et al.^224^ In their review, the authors excellently described the structure–function relationship and highlight the recent challenges and pointed out the crucial importance in the understanding of nanostructure and self-assembly of DESs. McDonald et al.^225^ have shown that using a methyl-terminated primary cation “switches on” the solvophobic self-assembly of cation alkyl chains in DESs to produce nanostructured solvents. Buzolic et al.^226^ discovered that an amphiphilic HBD, specifically C4 and C6 acids, can induce nanostructure formation in DESs. Furthermore, it was observed that the definition of the nanostructure improves with an increase in HBD chain length (Figure 4).

This paves the way for DES design, control of forces between ions, and the dissolution of more hydrophobic solutes. Additionally, such an approach provides a mechanism for the improvement of the functionality of DESs by incorporating properties related to nanostructures from their ionic liquid cousins while retaining their crucial advantages intrinsic to DESs (low cost and easy preparation). Amphiphilic nanostructured DESs will unravel their intrinsic properties with respect to synthesis bioinspired hybrid materials that are based on organic precursors (TEOS, TBOT, TIP, TEOG, etc.). In this respect, DESs amphiphiles can be employed to take advantage of (i) increasing solubility of organic precursors,^227^ (ii) DESs self-assembly into nanostructures with well-defined size and geometry but also to exploit (iii) their natural organization in ordered arrangements, which play a role of structure driving agents.^228^ The amphiphilic nature of DESs can also enhance enzymatic activity in enzyme-driven biomineralization studies.^229^

### 2.11 Impact of Water on DESs Properties

The majority of DES systems based on organic salts, like choline chloride and urea, were found to be extremely hygroscopic and when exposed directly to the atmosphere can absorb up to 20% (w/w) of water. Hence, it is difficult to generalize DES behavior since different combinations of HBA and HBD can result in DESs with different affinity for water. Therefore, many scientists agree with the fact that the influence of water on the properties of DESs as well as their application should be considered individually.^214,230^ Multiple studies unambiguously demonstrated that the addition of H_2_O rapidly changes the heterogeneous 3D structures of pure DESs, leading to alteration of physicochemical characteristics and their behavior. The experimental data gathered for DES–H_2_O systems, thoroughly summarized by C. Ma et al. in 2018, clearly indicate that the water content can exhibit a significant impact on density, viscosity, and electrical conductivity.^231^ Therefore, tailoring properties of DESs with water was proposed as a promising strategy to design for applications. Recent studies performed on choline chloride:urea DESs composed of dry components under moisture-free conditions have shown altered values of phase transitions and thermal instability. In this study, it was noticed that the very low water contents result in increased viscosity and decreased molecular mobility in the DES, making it less mobile, which directly influences the observed thermal properties.^230^ Additionally, neutron total scattering and empirical potential structure refinement studies for a mixture of choline chloride:urea (1:2) with water revealed that the nanostructure of the DES is retained at a remarkably high level of water (ca. 42% of H_2_O). However, above 51% of the hydration level, the DES–water mixture should be described as an aqueous solution of DES components. Therefore, the plausible phenomenon of transition from ionic mixture to aqueous solution upon the increase in the water content should be taken into consideration in the case of development of DESs in the field of bioinspired materials.^232^

### 2.12 Biocompatibility and Environmental Effects of DESs

The significance of solvent biodegradability, nontoxicity, and biocompatibility cannot be overstated in the realm of bioinspired materials. These materials are designed to emulate the extraordinary properties and functions observed in nature, presenting immense potential across diverse fields, including medicine. Therefore, ensuring the safety and compatibility of these materials is crucial to their successful translation into practical applications. Moreover, considering the increasing recognition of solvents’ impact on pollution, energy consumption, air quality, and climate change, the principles of green chemistry and sustainability^233^ demand a comprehensive understanding of solvent toxicity, biocompatibility, and environmental fate. Scientists must adopt a holistic approach, considering the implications of solvents on both human health and the ecosystem, to develop bioinspired materials that meet stringent environmental and ethical standards.^234^

DESs are commonly asserted to be environmentally sustainable, with their characteristics often described in terms of “low toxicity, high biodegradability, low cost, and sourcing from renewable feedstock.” Nevertheless, this broad assertion tends to be unquestioningly accepted in scientific papers that discuss novel advancements and applications of DESs. Furthermore, the perceived sustainability of DESs can vary considerably depending on the specific components present in the eutectic mixture. Nejrotti et al.^235^ recently provided a comparative, broad-spectrum review on the sustainability of widely employed DESs (or DES-like mixtures), sharing ChCl as their common HBA component. The toxicity of a DES could then be different from that of its components. Authors pointed out that the combination of components within a DES, even if they are individually nontoxic, can give rise to synergistic effects that alter the toxicity of the mixture. This can be attributed to the emerging interactions within the supramolecular structure of the eutectic mixture. According to Nejrotti et al.^235^ who holistically analyzed toxicity and biodegradability of DESs (simultaneously analyzing the impact of DESs production on the environment), the sustainability of the choline chloride (ChCl)-based deep eutectic solvent can be ordered as follow ChCl:glycine > ChCl:urea > ChCl:ethylene glycol > ChCl:malonic acid.

The results presented in several corresponding articles describing DESs toxicity^236,237^ clearly show that that the initial belief that DESs are universally environmentally friendly, solely based on the nature of their constituents, is somewhat simplistic. The findings highlighting the potential toxicity of DESs emphasize the need for rigorous toxicological assessments specific to bioinspired materials that will be dedicated for biomedical applications. These assessments should encompass comprehensive studies to understand the potential risks, identify safe exposure limits, and develop appropriate guidelines for the use of DESs in biomedical contexts. Consequently, there is an urgent necessity to thoroughly assess the toxicity of DESs and establish comprehensive and standardized protocols that align with their potential applications. The potential residues of DESs in bioinspired materials and its impact must be carefully analyzed. Indeed, the development of DESs-based bioinspired materials, including eutectogels intended for direct contact with biological systems like implantable medical devices or biocompatible coatings, require careful evaluation of DESs’ effects on cellular responses, tissue compatibility, and long-term biocompatibility. This thorough assessment is crucial to ensure the safety of patients and the efficacy of biomedical interventions. Holistic approach to DESs’ toxicity entails examining factors such as cell viability, proliferation, adhesion, and functionality when exposed to DES-based materials. Additionally, evaluating the compatibility of DESs with different types of tissues and their ability to integrate seamlessly within the biological environment is crucial. Conducting comprehensive evaluations of DESs’ biocompatibility ensures that these materials not only mimic natural systems but also exhibit the necessary biocompatibility and safety required for effective integration within the human body.

A distinguishing factor that unequivocally establishes the safety and environmental friendliness of DESs is their extremely low vapor pressure, which effectively eliminates the risk of atmospheric contamination and inhalation-related hazards.^235,238^ Lastly, DES implementation in industry also requires development of proper methods for solvent recovery and recycling and development of closed-loop systems and circular economy principles. By implementing efficient recovery techniques, the potential of DESs as environmentally friendly solvents can be fully realized.

## 3 Applications of Deep Eutectic Solvents

### 3.1 Biopolymer Processing

In various organisms, the formation of ordered arrays of inorganic crystals is facilitated through regulated nucleation occurring at the interfaces of the crystals and substrate biomacromolecules. Therefore, efficient processing of biomacromolecules is of crucial importance for the practical development of biomineralization-inspired hybrid materials. Chitin, collagen, and silk are identified as main biopolymers templating the biomineralization phenomenon. Chitin has been found in calcium carbonate^239^ and siliceous biominerals^240^ and collagen is a main organic component of bones^241^ and siliceous spicules of sponges,^242^ while chitin together with silklike proteins according to the famous Levi-Kalisman model^243^ regulate calcium carbonate formation in the bivalve mollusk shell. According to recent scientific reports,^241^ the importance of designing the biomineral-inspired materials is to understand the structural hierarchy as well chemistry of the biomineral on all length-scales.^244−246^ As biomineral deposition occurs on the nano, micro, and macroscale, by mimicking this process, researchers need to ensure efficient hybridization of the inorganic with selected biopolymers.^247^ This is not a trivial task if we consider, for example, that chitin is nonsoluble in the most known solvents.^248−250^ DESs are recently reported as a green, sustainable, and efficient chitin solvents and do not cause significant impact on the chitin crystallinity after its precipitation from DES.^251−254^ The hypothesized mechanism of chitin dissolution is explained as the interaction between the HBA and HBD components of a DES with inter- and intramolecular H-bonds of chitin that results in the breaking of the H-bond network of chitin and, as a result, facilitating its dissolution.^255^ This feature opens the new synthesis possibilities for creation of chitin-based bioinspired inorganic–organic materials. Among polysaccharides, cellulose is also widely used as a template for development of advanced biomineralization-inspired materials.^256−260^ Numerous reports describe the dissolution, nanofibrilation, and modification of cellulose in DESs,^261−267^ which consequently open an entirely new way for creating such composites.

**Figure 5:**
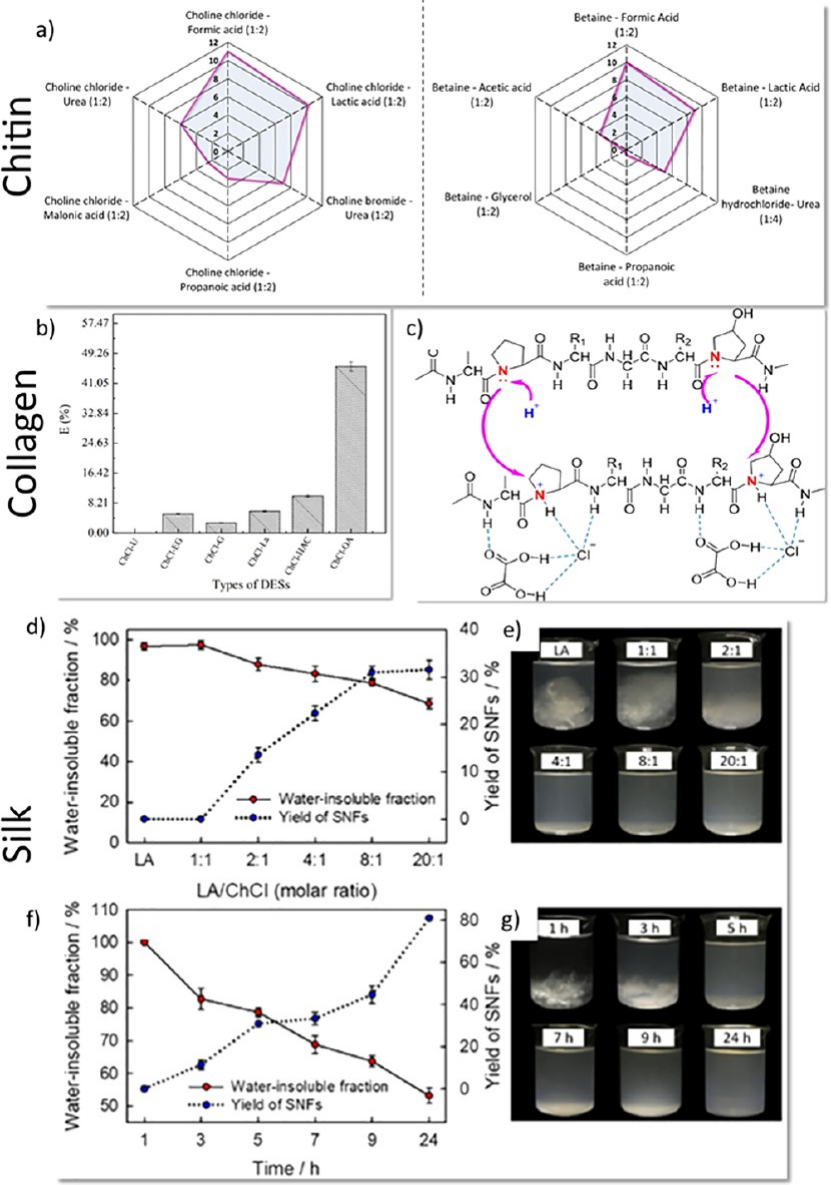
(a) Solubility of chitin (wt %) in selected DESs, according to ref (146). (b) Efficiency of collagen extraction from cod skin with respect to applied DES and (c) mechanism of interaction of DES with collagen. Reproduced from ref (272). Copyright 2017 American Chemical Society. Impact of the (d) DES composition and (f) treatment time on the water-soluble fraction and silk yield. The photographs of supernatants collected after DES treatment with various molar ratios of DES components (e) and various processing time (g). Reproduced from ref (273). Copyright 2020 American Chemical Society.

DESs have also been efficiently applied in the processing of chitin (Figure 5a), cellulose, collagen (Figure 5b,c), and silk (Figure 5d–g). Silk fibroins were recently explored in the design biomimetic biomineralization-inspired multilayered materials^268^ and functional biomaterials.^269^ With the assistance of computational simulation, the materials that arise from multiscale self-assembly and comprise silk nanofibrils (SNFs), hydroxyapatite (HAP), and chitin nanofibrils (CNFs) exhibit nacrelike structures. These structures possess mechanical strength and toughness that surpass that of the majority of natural nacre and nacrelike nanocomposites. We hypothesize here that biopolymer solubility in DESs can ensure their efficient hybridization with selected inorganic phases, and this inorganic–organic molecular recognition process will be beneficial for practical applications. It should be noted that inorganic precursors dissolved in DESs can be effectively stabilized, and this will ensure effective mineral deposition on various levels of structural organization. Stabilization of inorganic precursors is achieved through several mechanisms. One mechanism is the formation of hydrogen bonds between HBD and the precursor, which can prevent the precursor from undergoing unwanted reactions or decomposition. Another mechanism is the coordination of the precursor with the HBA. This can occur when the HBA contains a coordinating group, such as a carboxylate or amine, that can interact with the metal center of the precursor. This coordination can also stabilize the precursor and prevent unwanted reactions and can have an impact on the mechanisms of reaction with dissolved biomacromolecules.

Moreover, according to the green chemistry rules,^270,271^ scientific efforts should be shifted toward using solvation ability of DESs with respect to silk, collagen, cellulose, and chitin in materials chemistry. This will represent new, rational, low-cost, and efficient strategies for the combining of all these building blocks together with inorganic precursors and design and preparation of robust, hierarchical, and functional nanomaterials that will meet a variety of application requirements in modern technologies. Such an approach will reduce or remove the need for harmful chemical additives and energy-inefficient equipment.

However, the delay in implementing DESs in this case is due to the complex dissolution of biopolymers by DESs, which is affected by numerous factors such as viscosity, pH, and polarity. Thus, there is urgent need to find the DESs structure relationship with biopolymer-dissolving ability.

### 3.2 Electrospinning and Additive Manufacturing

**Figure 6:**
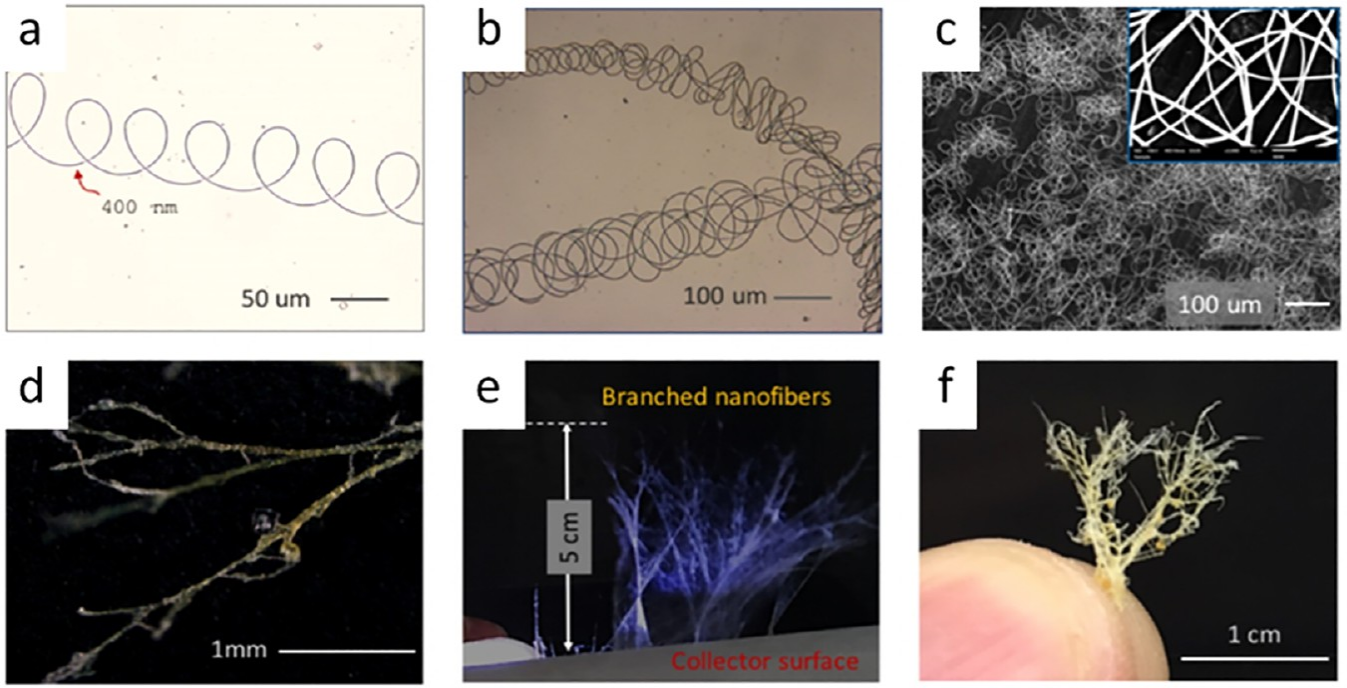
Tunability of fibers produced by electrospinning DES-Zein presenting (a) helical loops, (b) multiple helical loops, (c) multilayered nanofibers with (d) branches, (e) branched nanofibers, and (f) structures resembling cedar leaf morphology. Reprinted with permission under a Creative Commons Attribution (CC-BY) 4.0 from ref (276). Copyright 2020 Springer Nature.

Electrospinning is commonly employed as a straightforward and adaptable approach for producing bioinspired materials made of ultrathin nanofibers^274^ that allows control over various morphologies of the electrospun fibers. It has been already proven that the electrospinning of biopolymers from DESs lead to development of sophisticated materials with outstanding properties and morphologies. Application DESs in electrospinning eliminates the need for toxic or flammable volatile organic solvents (that are usually used for biopolymer processing). Additionally, most of the precursors and waste are designed to be recycled, therefore it fulfills the demand of sustainability and is a promising step torward zero-waste technology.^275^ Electrospinning of proteins using DESs enables unique control over the fiber morphology (Figure 6a–e). It has been observed that HBA in DESs preserve the α-helix configuration during electrospinning and leads to formation of unique coil-shaped fibers, and in some extreme examples electrospun fibers form sophisticated 3D architectures resembling the cedar leaf structure (Figure 6f).^276^

Surprisingly, there is a lack of reports reporting electrospinning of inorganic–organic biomineralization-inspired materials from ILs or DESs. This area needs urgent exploration as it can be a milestone in the development of biomimetic hybrid materials for water purification and enzyme immobilization. Obtained materials may have excellent mechanical properties that will extend their practical importance.

Another application of DESs is in 3D printing. 3D printing is a ground-breaking additive manufacturing technique already used in various advanced applications and broadening the prospects of a technology from nano to even industrial macroscale^277−280^ and could be envisioned to help synthesize biomineralization-inspired materials.

Bioinspired 3D printing of biomineralization-related or biomineralization-inspired structures is focused on (i) calcium phosphates^281^ because of their abundance in natural organisms and their compatibility with bone-implant applications^282^ or (ii) silica bioinspired structures with nanoscale precision.^283^ These materials also mimic the hierarchical structures found in native biominerals producing their unique mechanical properties such as high toughness.^284−289^ Considering that DESs can facilitate biopolymers dissolution, stabilization of inorganic precursors, and even can undergo polymerization, it is a natural consequence that the scientific community should shift their attention to explore the potential of DESs-based pastes that will combine biomacromolecules and inorganic precursors. By understanding the structure–property relations in biominerals, as synthetic chemists and materials engineers, it would be beneficial to use 3D-printing and a wider palette of building blocks (in contrast to natural organisms) that will enable the development of a new generation of biomimetic materials that will even outperform the natural structures. Therefore, much attention should be paid to the 3D-printing-assisted fusion of biopolymers, electroconductive DESs, and inorganic constituents (i.e., MXenes,^290,291^ MOFs,^292^ perovskites, POSS,^293^ M_*x*_O_*y*_^294−296^) that will mimic the structural hierarchy found in biominerals. Such a new fabrication technique combined with unique stability of DESs could enable the design and fabrication of the smart structures that are lightweight yet strong for biomimetically soft robotics.^297^ This unexplored direction will definitively require the comprehensive analysis and understanding of the flow behavior of above-mentioned biopolymers in DESs, which will open a new avenue for research that will have a fundamental impact on the additive manufacturing technologies.

### 3.3 Enzyme-Mediated Biomineralization in DES?

Recently various research groups^298,299^ confirm that DESs can be viable enzyme activators and stabilizers that lead toward more sustainable and energy efficient biotechnological processes. Huang et al. studied *Penicillium expansum* lipase performance in water solutions of 24 DESs. It has been found that by selecting a proper DES as an additive with an optimal salt/HBD ratio, an enzyme can be effectively activated and/or stabilized. The case of the choline acetate:glycerol (1:2) lipase showed a 1.4-fold increase in activity and a 17.4-fold enhancement in stability. However, the impact of DESs can be protein/enzyme-specific. The same DES induces different behaviors with different proteins/enzymes. Molecular dynamics simulations indicate that the intermolecular hydrogen-bonding network within DES components could effectively prevent the diffusion of DES components into the enzyme molecule, thereby decreasing the denaturation.^300^ However, findings reported by Cao et al.^301^ for nearly anhydrous DESs indicate that ChCl-acid DESs are not biocompatible solvents for enzymatic catalysis. They also found that impact of DESs on the enzyme activity and thermal stability is directly linked with the polarity, α and β parameters, and hydroxyl group content in DESs.^301^

This article is more focused on highlighting potential avenues for enzymatic-mediated synthesis of biomineralization-inspired materials. Therefore, for better understanding and insight toward enzyme stabilization and activation by DESs, readers are kindly referred to following review articles (refs (301−304)).

One advantage of using DESs as solvents for enzymes is their ability to stabilize enzymes in nonaqueous environments. Some of the reports, confirm that enzymes have higher activity in some DESs than in aquatic environments.^305^ This is crucial regarding studying biomineralization phenomenon in extremophiles (see section 3.5). Such an approach will enable one to take the full advantages and new opportunities offered by DESs. Especially when we consider that (i) choline chloride (component of most studied DESs) is an effective stabilizing agent of inorganic precursors,^306,307^ (ii) DESs effectively coordinate metal ions,^82^ and (iii) DESs have the potential to facilitate novel biocatalytic synthesis pathways that are not achievable in traditional reaction media such as aqueous buffers.^308^

Considering the utility of enzymes in biomineralization-inspired syntheses, it is essential to investigate how the enzymatic control over the mineral nucleation and crystallization is affected by different DESs.

### 3.4 DNA-Origami

DNA as a biomacromolecule has also been successfully used as template or mold in the bottom-up fabrication of well-defined mineralized nanostructures, ranging from nanometers to submicrometers, with sophisticated geometrical geometries.^309−313^ Recently, “scaffolded DNA origami” has become widely recognized as one of the most promising methods for assembling techniques and paving the way for unconventional synthesis approaches of nano-objects with tailored shape and functionality.^314^ In the laboratory, scientists can fold a long single DNA strand by DNA short strands (staple strand) that are complementary to the desired template strand. With the careful design, supported by computational molecular dynamics, the possibilities of DNA-origami are practically unlimited. The fundamental background knowledge regarding DNA-origami nanotechnology including origami design, synthesis, functionalization, and characterization is described in the comprehensive reviews:^314−317^ readers are strongly encouraged to follow these fundamental papers for a better overview of this extremely versatile technology.

**Figure 7:**
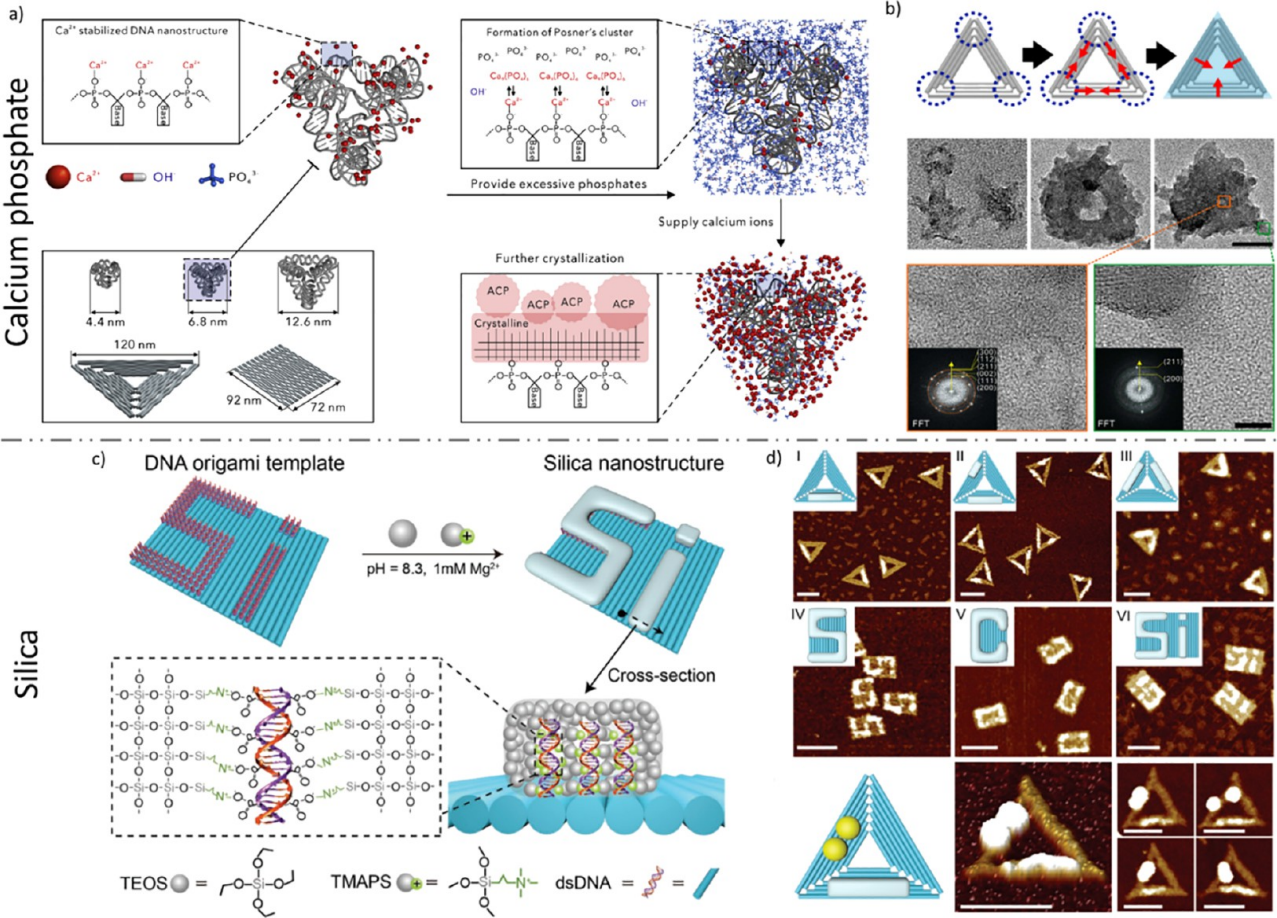
(a) Schematic representation of DNA-framework-templated crystallization of hydroxyapatite that involves DNA stabilization by calcium ions, formation of amorphous calcium phosphate due to local supersaturation, and further aging and crystallization of nanohydroxyapatite. (b) Transmission electron microscopy images showing the triangular DNA origami-hydroxyapatite nanocomposites with triangular morphology. Interestingly, the triangle tips center the crystallization trend. Reprinted with permission from ref (311). Copyright 2020 Elsevier. (c) Schematic representation on the proposed mechanism of DNA-origami silicification. (d) AFM imaging of various structures obtained experimentally. Reproduced with permission from ref (312). Copyright 2020 John Wiley and Sons.

This section is highlighting the potential of DNA-origami technology that provides a programmable platform for the development of hybrid, inorganic–organic hierarchical structures, inspired by the mostly distributed biominerals (calcium phosphates and silica). Last findings demonstrate that extracellular DNA functions as an initiator of collagen intrafibrillar mineralization.^318^ Recently, Liu et al.^311^ reported successful utilization of the DNA chiral scaffold for precise calcium phosphate crystallization that guides the mineralization of calcium phosphate with prescribed two-dimensional and three-dimensional shapes on the nanolevel (10–100 nm), Figure 7a,b.

It has been established that morphological features (size and shape) of DNA-calcium phosphate hybrid nanostructures are programmed through the utilization of structural information that is encoded within DNA sequences, in conjunction with electrostatic interactions between the DNA phosphate backbone and mineral counterparts (Figure 7a).^311^ Additionally, it has been found that DNA is able for atomic-scale control of calcium phosphate formation, due to the exceptional match between crystal plane distance calcium phosphate (hydroxyapatite, synthetic,^319^ as well biogenic^320^) along the *c* axis (002) and distance between adjacent base pair of DNA that is equal 0.34 nm.^311^ The biggest challenge in this method results from high density of phosphate groups in DNA that cause local calcium ions supersaturation and lead to relatively fast nucleation, crystallization, and uncontrolled overgrowth of hydroxyapatite nanocrystals, losing the shape of the DNA-template. Wu et al.^321^ in their recent study, overcome this issue by using the particle attachment method and using magnesium ions that stabilize DNA templates and slow down the spontaneous crystallization of hydroxyapatite.^322^ This allowed for the precisely controlled formation of the calcium phosphate layer with fine-tuned morphological features and functions through transcription of DNA-origami. Corresponding to the biominerals, obtained hybrids possess improved thermal stability and mechanical properties.

Customized 2D and 3D hierarchical silica-DNA hybrid nanostructures (with size 10–1000 nm) that replicate the wide range of geometric information on DNA-origami chiral scaffolds were successfully reported^309,312,323^ and are definitively a step forward in the mimicking of the complex geometrical architectures of natural silica-based biominerals like diatom frustules or skeletons of radiolarians (silicoflagellata). Liu et al.^323^ proved that the thickness of the amorphous silica (opal-like) layer deposited on the DNA surface can be simply tuned by adjusting the silicification time. Interestingly, the nanomechanical studies of formed DNA-silica hybrid structures possess ten-fold higher Young’s modulus than the DNA template while maintaining its original structural flexibility^323^ that are correspondingly found in naturally occurring biominerals.^324^ Shang et al.^312^ with use of a molecular dynamics simulation found that double-stranded DNA scaffolds possess electrostatic affinity to the positively charged silicic acid precursors. This was used as an advantage in development of a novel strategy to site-specific synthesis fabrication of nanostructured DNA-silica hybrids with nanoscale precision (Figure 7 c,d).^312^

Also, the DNA-templated metal (gold, silver, copper) nanoclusters mimicking the extreme biomineralization (for review, see ref (3)) are now emerging as a new type of functional nanomaterial with a wide spectrum of unique applications in modern technologies.^325^ All these examples clearly demonstrate that the DNA-origami technique is a groundbreaking nanofabrication strategy that opens a new avenue for bioinspired materials science and precise, programmable bottom-up inorganic material synthesis. However, a potential limitation of DNA-origami nanotechnology is the fact that it is mostly limited to aqueous or substantially hydrated media; application of organic solvents usually causes alterations of the DNA helical structure or the loss of base pairing.

**Figure 8:**
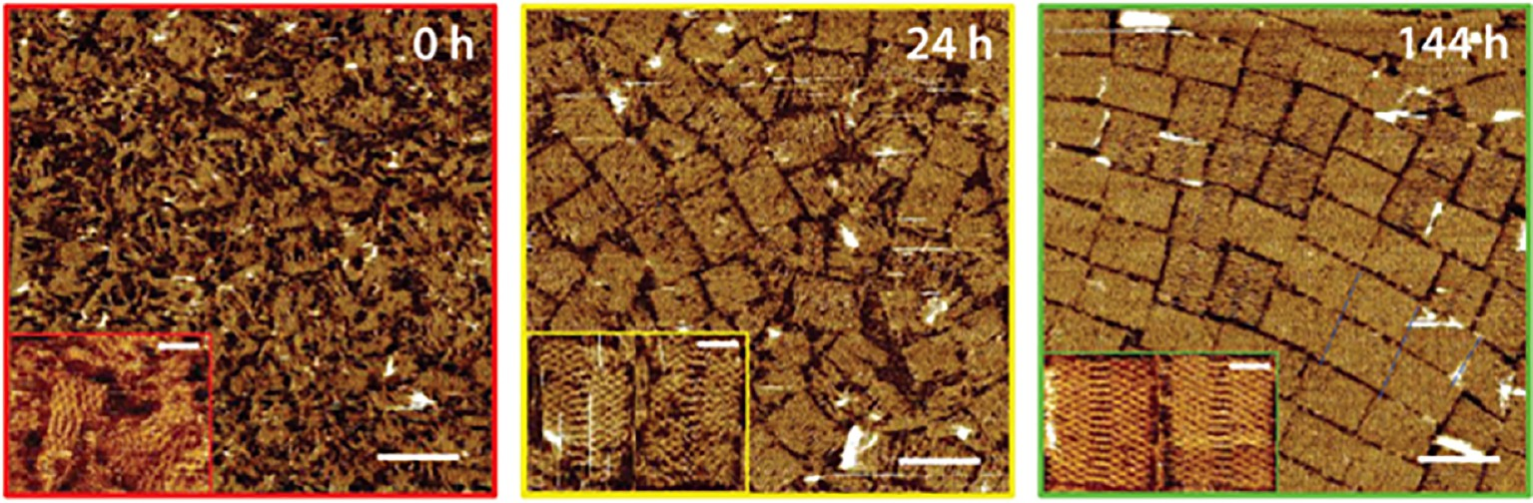
AFM images showing the DNA origami folding in DES (choline chloride-glycerol, 1:4) at 20 °C at selected time points. Reprinted with permission from ref (326). Copyright 2015 John Wiley and Sons.

Recent advances in this field done by a research group headed by Professor Nicholas V. Hud proved that anhydrous DESs composed of choline chloride:urea (1:2 molar ratio) and chloride chloride:glycerol (1:4) are suitable environments in which DNA can form stable forms of duplex, triplex, and G-quadruplex structures.^326^ It was observed that DNA maintains B-form helical structures in DES, and the regenerated DNA shows a high thermal and pH stability. According to Gállego et al.,^326^ a plausible mechanism for DNA solvation involves the interaction of choline cations with the DNA phosphate backbone (Figure 8).

Additionally, given previous reports that DESs support both enzymatic catalysis and controlled crystallization of inorganic nanostructures, the possibility that catalytic nucleic acids and enzyme–DNA complexes could be used as a tool for development of biomineralization inspired materials and better understanding of biomineralization. Bringing the exquisite control of DNA through DESs paving the way for development of new approaches in the synthesis nanostructured bioinspired materials and their efficient application in the new generation of nanoelectronics, nanophotonics, catalysis and many other areas improving the quality of human life.

### 3.5 Extreme Biomimetics

The impressive capacity of living beings to adapt and thrive in harsh or extreme environments has captivated and inspired numerous scientists from various research disciplines. Exploration of the mechanisms underlying biomineralization strategies in extremophiles and transferring this knowledge into laboratory practice in the development of new hybrid materials has become a fundamental driving force for the development of recent extreme biomimetics.^327−329^ This direction, pioneered by Prof. Hermann Ehrlich in 2010, is now a vibrant area of research that led to the synthesis of a new generation of biomaterials and biocomposites that are characterized by unusual and rather unexpected functional properties.^330,331^ Extreme biomimetics encompasses a range of subjects, including the study of biosilicification in Antarctic diatoms that thrive in temperatures below −20 °C, the examination of diverse biomineralizing organisms that inhabit the freezing point of seawater (−1.9 to 4 °C), and the investigation of fauna found in hydrothermal vents and hot springs with temperatures ranging from 60 to 121 °C.

Gaining a comprehensive understanding of the fundamental principles of biomineralization that allow living organisms to thrive in extreme environments has the potential to pave the way for the development of innovative principles of extreme biomimetics. By applying these principles in various technological applications, researchers can harness the remarkable adaptive mechanisms of living organisms and improve the performance of materials and devices in harsh conditions.

Recent findings published by Gertrudes et al.^66^ revealed that metabolites of some extremophiles are forming natural deep eutectic solvents (NADES) that play the role as cryoprotectants in the cells. NADES are also responsible for dissolution, transport, and biotransformation of poorly water-soluble molecules, improving the activity and stabilization of enzymes. This might be a missing key to understanding the puzzling biomineralization (calcification and silicification) phenomenon in psychrophilic organisms. Detailed studies of biosilicification, biocalcification, and iron- or manganese biomineralization in psychrophilic organisms are of high importance to close the gap in knowledge about this mysterious phenomenon. We strongly believe that a deep insight into the role of DES/NADES in stabilization of biomacromolecules (chitin,^332^ collagen,^242^ ferritin,^334,335^ actin^336^); enzymes (silicatein,^337−339^ glassin^340,341^); and inorganic precursors (silicic acid or ACC, iron, or manganese ions) will shine new light on the psychrophilic biomineralization pathways. As a result, this will trigger new research oriented on the development of a new class of biomaterials, with unusual properties, according to extreme biomimetics philosophy.^342^

Open questions: (i) Can deep eutectic solvents act as stabilizers for inorganic precursors (silicic acid, ACC, phosphate, and metal ions)? (ii) How do deep eutectic solvents affect the stability of enzymes (e.g., silicatein, urease, phosphatase) and proteins (e.g., collagen, silk, actin) that are identified as responsible for biomineralization phenomenon? (iii) What is the DESs structure–solvation relationship with respect to the chitin, silk, collagen? Which mechanism underlies the solubility of biomacromolecules in deep eutectic solvents? (iv) How do DESs impact the structural peculiarities and dynamics of proteins in terms of molecular and fibrillar levels? (v) How do DESs nanostructures guide the synthesis of biomineralization-inspired materials? (vi) How can predictive multiscale modeling be utilized effectively, especially generative deep learning, to discover new compounds including an assessment of synthesizability?

## 4 Computational and Artificial Intelligence Perspectives

The development of next-generation biomaterials, using DESs, biopolymers (chitin, collagen, silk, DNA), enzymes, and inorganic precursors, must incorporate the principles of green chemistry and engineering into a broader definition of performance, which includes sustainability considerations. Achieving this ambitious aim will require modern tools such as computational multiscale modeling and artificial intelligence. This synergy will inform predictions on how even subtle modifications on the molecular scale chemistries or nanostructural organization of DESs will impact the final product in terms of its properties on the macroscale such as viscosity or self-assembly.

One approach to such predictions is the use of molecular dynamics (MD) to build large-scale models that simulate dissolving biopolymers in DESs.^343,344^ MD and related quantum scale techniques such as density functional theory (DFT) have been used to study biomass, and biomaterials will complement this work and can provide direction for further research.^343−346^ Quantum-level (e.g., DFT) and atomistic or coarse-grained MD simulations could produce data that describes the behavior of numerous solvents and biopolymers without performing time-consuming manual experiments. By using computational methods to simulate the dissolution of biopolymers in DESs, we can rapidly gather data on structure–property relationships that can better inform our procedures in preparing pure biopolymers for bioinspired materials synthesis.

**Figure 9:**
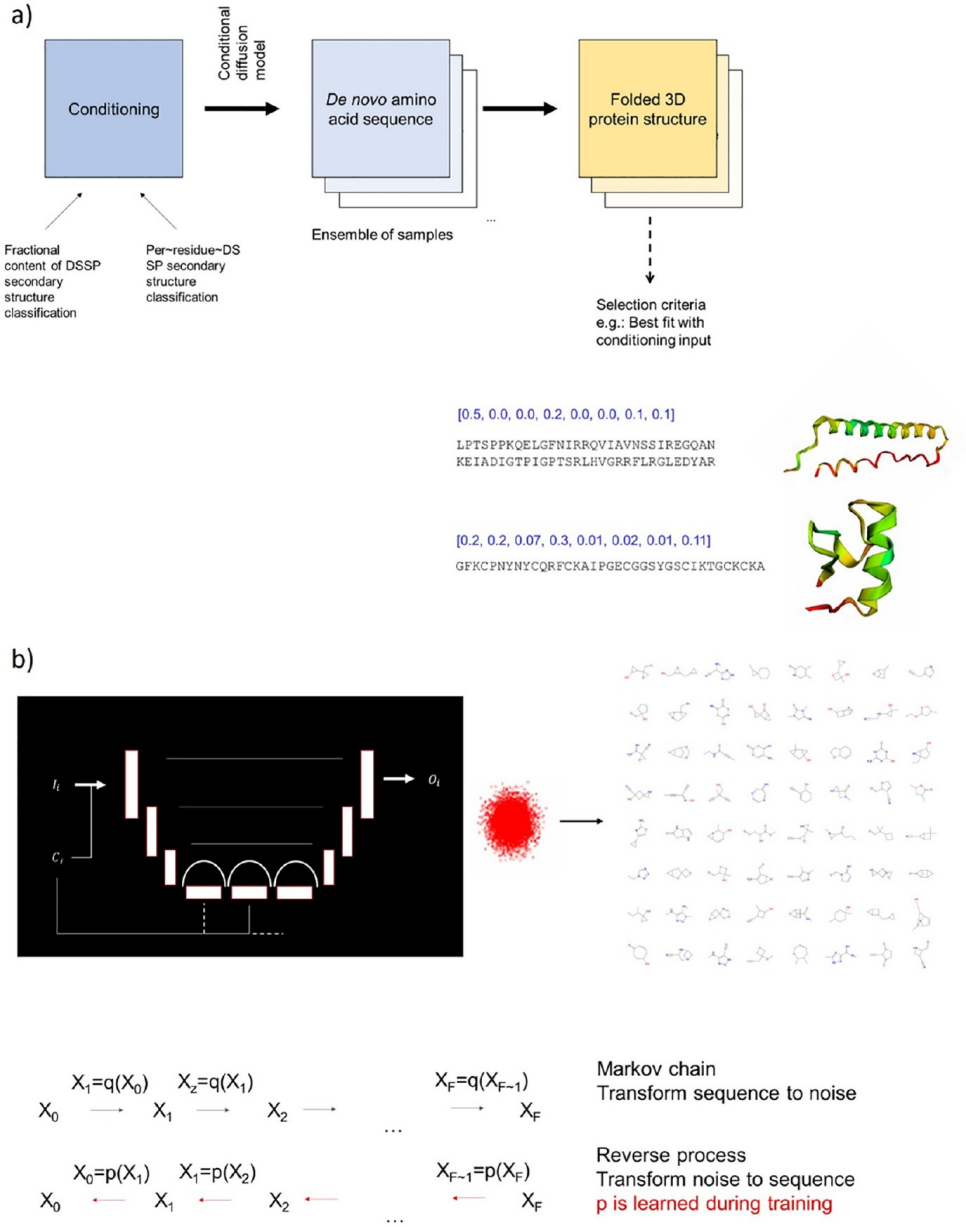
(a) Overview of generative deep learning models that use secondary structure design objectives to predict amino acid sequences and 3D protein structures. These models can operate on either the overall content or per-residue structure of the protein. (b) Depiction of the 1D U-net architecture that translates an input *Ii* into an output *Oi* under a condition set *Ci*. This model takes a conditioning description of the desired secondary structures as input and produces various sequences of AAs from random noise vector sources and illustration of the Markov chain of noising (top) and denoising (bottom). Reprinted with permission from ref (97). Copyright 2021 Elsevier.

Artificial intelligence approaches, especially machine learning (ML) and deep learning (DL) algorithms, can also be used to discover new combinations of DESs for a variety of applications. ML is emerging as a powerful tool in the fields of chemistry,^347^ materials and biomaterials science,^348−352^ and mechanical engineering,^353−357^ attributed to its power to predict materials properties, design *de novo* materials,^358^ and discover new mechanisms beyond intuitions.^348^ For DESs ML has primarily been used to estimate density and viscosity.^184,195^ In the cases of predicting density, researchers have applied supervised machine learning techniques and have quantified the accuracy of seven different machine learning methods before determining their highest accuracy tool. For viscosity, another group has applied multiple machine learning algorithms to train a model that can achieve strong predictive performance.^195,359,360^ The success of these past models further begs the need to advance in this direction of computational approach. Another avenue of computational work would be to utilize generative modeling. Generative neural network models can also be used to generate *de novo* protein designs (Figure 9) with specific mechanical behaviors by coupling it with coarse grained analysis and has been demonstrated to solve the inverse problem of correlating and tuning polycrystalline materials to mechanical properties in which this framework can be used to guide *de novo* biomaterial design.^361^

Similar approaches to what has been accomplished previously can be taken by studying DESs. A model could be developed that is capable of both understanding DESs and even discovering new DESs. Recently, we reported a series of deep learning models to solve forward and inverse design problems in molecular modeling and design.^362^ Using both diffusion models inspired by nonequilibrium thermodynamics and attention-based transformer architectures implemented via a generative language model, we demonstrate a versatile framework capable of capturing complex chemical structures from commonly used simplified molecular-input line-entry system (SMILES) input. Trained jointly in tasks related to the quantum machines (QM9) data set and DESs, the model can predict various quantum mechanical properties and critical properties to achieve deep eutectic solvent behavior. Several potential combinations of DESs are proposed based on this framework, i.e., monoethylcholine chloride and 4-methylcatechol.^362^

**Figure 10:**
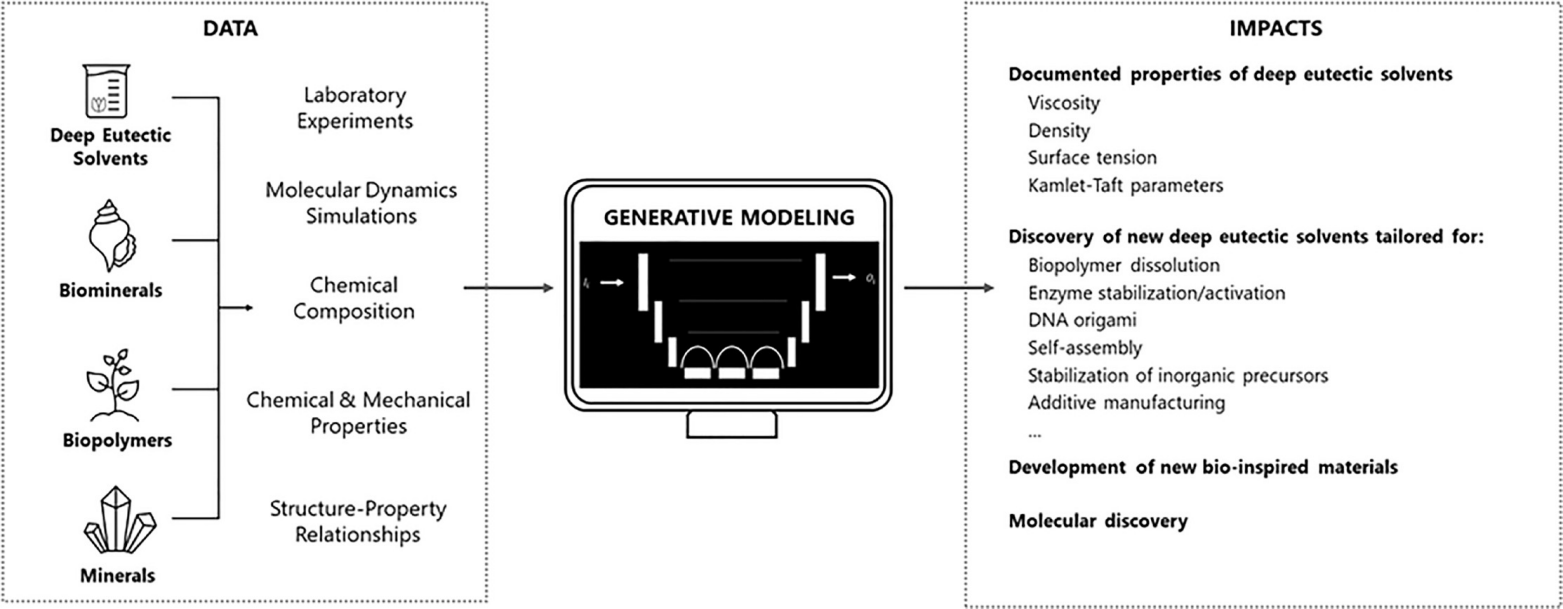
Schematic illustration of the expected impact of using machine learning techniques in the DESs-assisted development of bioinspired hybrid materials synthesis. The potential data set includes results from laboratory experiments, developing synthetic data using molecular dynamics simulations, and a data set on structure–property relationships. The data set may span DESs, biominerals, biopolymers, and minerals. The use of generative models may provide the field with insights into novel material discovery for specific applications and material properties.

Further examples of inverse problems that can be addressed are which DESs would best dissolve biopolymers or aid the creation of a specific desired biomineral hybrid material. In order to develop generative models for this purpose, more experimental data on DESs polymer solubility, inorganic crystallization (growth rate) in DESs would significantly support progress. The creation of future versions of generative models centered on DESs would then greatly aid in bioinspired materials synthesis and biopolymer organic–inorganic hybrid synthesis (Figure 10).

It is important to note that high-throughput data set collection is crucial with machine learning. Parameters of interest might be SMILES, melting point, density, viscosity, deep eutectic melting temperature, and the Kamlet–Taft parameters. Experimental data sourced from the literature on DESs can be gathered and cleaned for data set creation. Additionally, experimental data can be produced in which the procedures of experimentation will be openly documented and public.

With readily available data sets, models can be trained and tested with this experimental data serving as ground truth, combined with inputs from simulation data from molecular dynamics or DFT calculations.

Applying computational methods toward studying DESs is a high potential area of research that would accelerate innovation in the fields of biomimetic chemistry and materials science.
